# Staying Home, Distancing, and Face Masks: COVID-19 Prevention among U.S. Women in The COPE Study

**DOI:** 10.3390/ijerph18010180

**Published:** 2020-12-29

**Authors:** Katherine M. Anderson, Jamila K. Stockman

**Affiliations:** 1 Department of Medicine, Division of Infectious Diseases and Global Public Health, University of California San Diego, La Jolla, CA 92093, USA; k4anderson@health.ucsd.edu; 2 Department of Behavioral, Social, and Health Education Sciences, Emory University Rollins School of Public Health, Atlanta, GA 30322, USA

**Keywords:** COVID-19, prevention and control, SARS-CoV-2, United States, women, logistic models

## Abstract

The novel coronavirus (COVID-19) pandemic has significantly impacted United States residents. Prevention behaviors are critical to minimizing transmission of SARS-CoV-2 in the U.S., to ultimately reduce the health, social, and economic burdens of COVID-19. Yet, health behavior decision-making is complex, and uptake of preventative behaviors has been variable. Women may provide pro-prevention behavior modeling to their networks, facilitating uptake diffusion. The COPE Study enrolled 491 women residing in the United States from May to June of 2020; women completed an online survey of COVID-19 experiences and prevention behaviors. We employed binary logistic modeling to identify factors predicting women’s practice of (1) staying home except for essential activities, (2) physical distancing in public, and (3) wearing a face mask in public. Findings demonstrate that women’s prevention behaviors are influenced by multilevel factors. Women living in urban environments, having minimal formal education, or having a household annual income of USD 30,000–50,000 are less likely to practice prevention behaviors. Cultural context may be an important factor in the decision-making process. Results aid in the identification what interventional “levers” may warrant consideration to promote uptake of such behaviors, and whom to engage. Because women are modelers of behavior, it is critical to engage them in prevention behavior interventions.

## 1. Introduction

The novel coronavirus (COVID-19) pandemic, caused by the virus SARS-CoV-2, has required vast alterations to daily life in the United States (U.S.) in an effort to reduce transmission, morbidity, and mortality. The U.S. has seen one of the worst documented epidemics worldwide, with over 19 million cases and 300,000 deaths [1]. In response, national and international agencies have promoted evidence-based prevention behaviors to reduce SARS-CoV-2 transmission [2].

Chief among the recommended prevention behaviors are staying home except for essential activities, physical distancing of at least six feet in public, and wearing a face mask in public [3]. Staying home is the most efficacious of these behaviors [3,4], while the effectiveness of physical distancing and masking may be significantly limited by insufficient distancing and incorrect mask use [3]. Practice of prevention behaviors has been inconsistent in the U.S. [5,6,7,8]. A May 2020 CDC survey found that while over 90% of U.S. adults had been to a public area in the previous week, 77% reported staying home except for essential activities; approximately 60% “always” physically distanced in public, and 60% “always” wore a face mask in public [6]. Earlier assessments had found 50% of U.S. adults wore face masks in public, though 87% physically distanced [5]. Some research indicates less or differential compliance among younger, less financially secure, male, and/or Black individuals [5,6,8].

Early research has explored the pro-prevention behavioral impacts of personal and social factors [9,10,11,12]. These include differential political messaging, in which geographic areas with a high population of politically conservative individuals engage in less physical distancing, and maintain differential beliefs regarding COVID-19 risk from their politically liberal counterparts [9]; high perceived susceptibility and severity of COVID-19, self-efficacy to practice prevention behaviors, and perception that doctors play a powerful role in the continuing epidemic as associated with adherence to CDC-recommended prevention behaviors [10]; fear of COVID-19 as a positive predictor of prevention behavior change [11]; and, COVID-19 risk perception and trust in science as predictors of compliance with prevention guidelines, with mediation by political conservatism, religious orthodoxy, intellectual curiosity, and conspiracy ideation [12].

Yet, health behavior decision-making is nuanced, with myriad factors influencing each health behavior decision. Individuals operate within systems that influence behavior [13], including but not limited to personal factors that are perceived and reacted to by society (i.e., race/ethnicity and racism or xenophobia, education/income and classism), interpersonal relationships, familial organization and other organizational structures, and community or environmental context. Such multilevel influences are described in the Social Ecological Model, from which the current levels of influence are derived [14]. Identification of interventional targets within these domains can allow for activation of mechanisms of health behavior change, to increase prevention behaviors. In the endeavor of promoting prevention behavior, women are a key population; they are often highly influential in health seeking behaviors for their familial unit, including for children [15,16,17,18] and partners [19,20,21]. Modeling of pro-prevention behaviors for those around them may lead to a spillover effect of increased behaviors within their social network and community.

Given the variability in compliance with public health prevention guidance and the keystone role of women, identification of interventional targets among women in the U.S. is critically needed for uptake of prevention behaviors, to ultimately reduce the health, social, and economic burdens of COVID-19. With the likelihood of a lasting COVID-19 epidemic in the U.S., and with consideration for the possibility of future epidemics, the current analysis seeks to examine the personal-level, interpersonal-level, organizational-level, and environmental-level factors associated with three key prevention behaviors among a sample of U.S. women as behavioral models, so as to inform current and future epidemic responses.

## 2. Materials and Methods

From May to June of 2020, we deployed The COPE Study, a cross-sectional survey of experiences related to COVID-19 and COVID-19 prevention behaviors among adult women residing in the U.S. The COPE Study was approved by the University of California San Diego Human Subjects Protection Program Institutional Review Board (#200663). Eligible participants self-identified as women, were aged 18 or older, resided in the U.S., and were able to understand English. Women were recruited using Facebook Advertising, wherein women aged 18 and older were targeted for advertisements on Facebook (83.1%), Instagram, and other sites deemed relevant by the Facebook Advertising algorithm (“Audience Network” 16.5%). Approximately 93% of individuals who entered the survey provided informed consent and responded to screening questions (633/682). Of 626 eligible women, 491 (78%) completed the self-administered online survey, and were emailed a USD 20 Amazon gift card in compensation. This research received funding from the University of California San Diego Office of Research Affairs Office for Equity, Diversity, and Inclusion.

Personal-level covariates included age, geographic region of residence, educational attainment, employment status, relationship status, number of children, household occupancy, urbanicity/rurality, and household income. Race/ethnicity was categorized as White; Asian, Native Hawaiian or other Pacific Islander (API); Black; Hispanic, Latina, or Spanish origin; American Indian or Alaskan Native (AI/AN); or, multiracial or some other race, ethnicity, or origin. COVID-19 specific individual-level covariates included experiences of coronavirus symptoms, knowing where to get tested, testing history, diagnosis/hospitalization history, and fear of COVID-19 [22]. We utilized the Fear of COVID-19 Scale, a 7-item self-reported measure of participants’ fear of COVID-19 [23]. Example items include being afraid of losing one’s life because of COVID-19, being unable to sleep because of worrying about getting COVID-19, and experiencing physical symptoms of fear. Responses followed a 5-point Likert scale ranging from “strongly disagree” to “strongly agree.” Responses were summed across all 7 items, with scores ranging from 7 to 35 (Cronbach’s alpha = 0.90).

Interpersonal-level covariates included relationship status and knowing if close others (e.g., family, friend, coworker) were diagnosed, hospitalized, or died from coronavirus. Organizational-level covariates included household size, composition, and income, while environmental-level covariates were U.S. region and urbanicity/rurality of residence.

Outcome variables were three prevention behaviors: (1) staying home except for essential activities, (2) physical distancing of six feet from non-household members, and (3) using a face mask in public [18]. To capture prevention behaviors, participants were asked “Which of the following prevention behaviors have you been using?” with the option to check each applicable behavior, indicating “yes”, or not check the behavior, indicating “no”.

We computed descriptive statistics for all variables, reporting medians and interquartile ranges (IQRs) for non-normally distributed continuous variables and frequencies and proportions for categorical variables. Chi-square tests, independent sample t-tests, and Mann–Whitney U tests were used to assess differences in each of the three prevention behaviors across independent variables. Independent variables were selected based on theoretical significance and mapped onto levels of the Social Ecological Model [10]. Due to the exploratory nature of this analysis, and the intent to identify factors associated with prevention behaviors based on theoretical classification and with the aim of informing interventions, we chose to include independent variables that were significant at *p* < 0.25 at the bivariate level in modeling, so as to not prematurely exclude factors that may have statistical significance in models and practical interventional use [24]. In analyses based on theory, potential theoretical significance of hypothesized relationships is often considered priority above statistical significance, supporting this decision [25]. Independent variables were assessed for collinearity, with no relationships identified that were both significant and had a Pearson’s correlation coefficient with a magnitude greater than 0.3. Given the absence of significant moderate or high correlation, and the exploratory nature of the present analysis, all variables were included in preliminary models. Binary logistic regressions were utilized to model each outcome variable using stepwise backward elimination to obtain a parsimonious model with predictive ability; all variables significant at the bivariate level were entered into the model, followed by the removal of the variable or set of dummy variables with the highest *p*-value in each progressive model. Models in which all variables, or at least one variable within a set of dummy variables, were significant at cutoffs of *p* < 0.10 and *p* < 0.05 [24,26,27] were presented given the potential for meaningfulness. Reporting of variables for such models follows the format: (Model 2; Model 3). All models demonstrate good fit and significance at a level of *p* < 0.001 using the chi-square goodness of fit test. All statistical analyses were performed using SPSS, version 26 [28]. Demographic characteristics for participants (*n* = 491) are presented in Table 1.

## 3. Results

### 3.1. Bivariate Analyses

At the bivariate level, each outcome variable was significantly associated with variables from all four domains (personal, interpersonal, organizational, environmental) at *p* < 0.25 (Table 2). Staying home was associated, with varying directionality, with (1) personal domain factors of race/ethnicity (*p* = 0.051), education (*p* = 0.200), employment (*p* = 0.164), age (*p* = 0.136), having had symptoms of COVID-19 (*p* = 0.15), having been tested for COVID-19 (*p* = 0.237), knowing where to get tested for COVID-19 (*p* = 0.042), and fear of COVID-19 score (*p* = 0.005); (2) interpersonal domain factors of relationship type (*p* = 0.005), knowing someone who was diagnosed with COVID-19 (*p* = 0.042), and knowing someone who died of COVID-19 (*p* = 0.148); (3) organizational domain factors of living with others (*p* = 0.005), number of participants’ children under 18 staying in household (*p* = 0.227), and annual household income (*p* = 0.001); and, (4) at the environmental level, urbanicity (*p* = 0.006).

Physical distancing was associated, with varying directionality, with (1) personal factors of race/ethnicity (*p* = 0.0921), education (*p* = 0.004), having a chronic disease (*p* = 0.054), knowing where to get tested for COVID-19 (*p* = 0.001), having been diagnosed with COVID-19 (*p* = 0.101), and fear of COVID score (*p* = 0.072); (2) interpersonal factors of relationship type (*p* = 0.004) and knowing someone diagnosed with COVID-19 (*p* = 0.032); (3) organizational factors of number of participants’ children under 18 staying in household (*p* = 0.074) and annual household income (*p* = 0.006); and, (4) within the environmental domain, urbanicity (*p* = 0.012).

Wearing a face mask was associated, with varying directionality, with (1) personal domain factors of education (*p* < 0.001), employment (*p* = 0.022), participants’ number of children under the age of 18 (*p* = 0.220), participants’ number of children aged 18 or older (*p* = 0.052), having a chronic disease (*p* = 0.139), and knowing where to get tested for COVID-19 (*p* < 0.001); (2) interpersonal factors of relationship status (*p* = 0.001) and knowing someone diagnosed with COVID-19 (*p* = 0.163); (3) organizational factors of number of participants’ children 18 or older staying in household (*p* = 0.163) and annual household income (*p* = 0.001); and, (4) environmental factors of region of residence (*p* = 0.023) and urbanicity (*p* = 0.004).

### 3.2. Outcome 1: Staying Home Except for Essential Activities

Table 3 presents the modeling findings for staying home except for essential activities. The initial model (Model 1), inclusive of all variables significant at the bivariate level, is available in Supplemental Materials. At the personal level, Latinx ethnicity, age, and fear of COVID-19 were included in Model 2. Interpersonal-level significant variables included being in a committed relationship but not married, and knowing someone who was diagnosed with COVID-19. Income under USD 30,000 was the only organizational-level variable included in the model, while urbanicity was the only environmental-level variable. In Model 3, all variables were retained except age and knowing someone who had been diagnosed with COVID-19. Both models demonstrate that Latinx women are at least 70% less likely to stay home except for essential activities compared to White women (OR: 0.220, *p* = 0.003; OR: 0.287, *p* = 0.011). Fear of COVID-19, though significant, only increased the odds of staying home by 7–8% (OR: 1.079, *p* = 0.004; OR: 1.075, *p* = 0.005). Women in a committed relationship, but not married, were six to seven times the odds of staying home compared to non-partnered women (OR: 6.819, *p* = 0.015; OR: 7.095, *p* = 0.005). Annual household income under USD 30,000 increased the odds of staying home by 4–5 fold (OR: 4.725, *p* = 0.001; OR: 4.317, *p* = 0.001), while annual income over USD 50,000 resulted in 3.5 times increased odds of staying home (OR: 3.473, *p* = 0.004; OR: 3.495, *p* = 0.003), compared to an annual household income of USD 30,000–50,000. Living in an urban environment decreased the odds of staying home by almost 60% (OR: 0.400, *p* = 0.017; OR: 0.421, *p* = 0.021).

### 3.3. Outcome 2: Physical Distancing in Public

The second outcome modeled was physical distancing in public (Table 4). The initial model (Model 1), inclusive of all variables significant at the bivariate level, is available in Supplemental Materials. Personal factors retained in Model 2 included race/ethnicity, educational attainment, having a chronic disease, and having been diagnosed with COVID-19; the only interpersonal factor retained was being partnered but not married; no organizational variables were retained in Model 2, and living in an urban environment was the only environmental-level variable retained. All variables were retained for Model 3 except for urbanicity, resulting in a final model with only personal and interpersonal factors. Compared to White women, women identifying as API had a 3–4 higher odds of distancing in public (OR: 4.323, *p* = 0.027; OR: 3.632, *p* = 0.047). In comparison to those who completed high school, a GED, or less, women who had some trade or vocational school or college had 4 times the odds of physically distancing (OR: 3.983, *p* = 0.028; OR: 4.044, *p* = 0.025). Women with a chronic disease were over 3 times more likely to physically distance (OR: 3.103, *p* = 0.012; OR: 3.334, *p* = 0.008). Conversely, women who had been diagnosed with COVID-19 had 95% lower odds of physically distancing (OR: 0.069, *p* = 0.004; OR: 0.052, *p* = 0.002). Married women had approximately 2.5 times the odds of practicing physical distancing compared to non-partnered women (OR: 2.285, *p* = 0.036; OR: 2.662, *p* = 0.011). While not retained in Model 3, women living in an urban environment had 48% lower odds of physically distancing in public than women living in nonurban communities (OR: 0.522, *p* = 0.054).

### 3.4. Outcome 3: Wearing a Face Mask in Public

Table 5 presents models for wearing a face mask in public. The initial model (Model 1), inclusive of all variables significant at the bivariate level, is available in Supplemental Materials. Personal factors included in Model 2 were education and knowing where to get tested for COVID-19. No interpersonal variables were retained. Organizationally, annual household income was retained, as were region of residence and urbanicity within the environmental domain. In Model 3, all variables were retained except for region of residence. Models indicate that women with some trade or vocational school or college had 3.5 times the odds of wearing a face mask compared to those with a high school diploma, GED, or less (OR: 3.455, *p* = 0.007; OR: 3.562, *p* = 0.005), while those with some graduate school or more had approximately 4.5 times the odds (OR: 4.435, *p* = 0.001; OR: 4.454, *p* = 0.001). Knowing where to get tested for COVID-19 increased the odds of masking by two-fold (OR: 1.967, *p* = 0.014; OR: 2.00, *p* = 0.010). Women with an annual household income USD <30,000 were over twice as likely to mask (OR: 2.284, *p* = 0.016; OR: 2.156, *p* = 0.022), as were women in households making USD >50,000 annually (OR: 2.25, *p* = 0.013; OR: 2.184, *p* = 0.013), compared to those making USD 30,000–50,000. Living in an urban community decreased the odds of masking by about 60% (OR: 0.433, *p* = 0.003; OR: 0.41, *p* = 0.002).

## 4. Discussion

In the present study of factors associated with COVID-19 prevention behaviors among U.S. women, we found that the practice of prevention behaviors varied widely across personal-, interpersonal-, organizational-, and environmental-level factors. These factors indicate (1) who to focus interventional efforts on, and (2) what experiences or characteristics may serve as “levers” for behavioral change.

### 4.1. Race/Ethnicity

We found differences in uptake of prevention behaviors by race/ethnicity. Latinx women had lower odds of staying home than White counterparts. Racial/ethnic minorities are overrepresented among essential workers, limiting their ability to work from home [29,30], which may limit the perceived urgency or feasibility of staying home for nonessential activities. Further, overrepresentation of racial and ethnic minorities in public-facing essential positions may be responsible for these relationships, as race and ethnicity themselves do not dictate behavior; rather, behavior is derivative of the social context that may be associated with race or ethnicity. Alternatively, racial/ethnic minority women may be more likely to live in environments not conducive to staying home, including multigenerational housing with a high number of residents [31,32]. Given the significant disparities in Latinx outcomes for COVID-19 [33,34], this population should be prioritized for interventions. Conversely, women identifying as API had significantly higher odds of physically distancing in public than White women. Explanations may include access to information and education regarding COVID-19, cultural practices regarding disease prevention, or performative practice of prevention behaviors in response to xenophobia; since identification of the origin of SARS-CoV-2 as China, API individuals have faced discriminatory backlash [35]. While beyond the scope of the current analysis, future research should assess possible interaction between race/ethnicity and other independent variables.

### 4.2. Education

Education was significantly impactful for two out of three prevention behaviors. We found increases in the odds of distancing and wearing a mask by 3.5–4 times among those who had some trade or vocational school, or some college, compared to those with a high school education or less. Further, women who had some or completed graduate school education had approximately 4.5 times the odds of wearing a face mask in public than women with a high school education or less. Given correlations between education and health literacy, this is not surprising [36,37], but documents the need for health literacy outreach among low educational attainment women. Further, this may be representative of environmental conditions of jobs requiring lower educational attainment, which may limit the ability of workers to physically distance. Staying home as a prevention behavior did not significantly vary by educational attainment, which may be reflective of this being the most convenient prevention behavior, or the one that is most difficult due to competing priorities unrelated to educational attainment and its social correlates, despite also being the most effective prevention behavior [3,4].

### 4.3. Personal and Interpersonal Experiences with COVID-19

Women who knew where to get tested for COVID-19 had twice the odds of masking in public, presenting testing education as a possible interventional “lever.” Those who are already practicing prevention behaviors may know where to get tested due to awareness of COVID-19, or knowing where to get tested may increase perceived susceptibility to COVID-19, prompting prevention behaviors. This relationship should be further explored to better understand directionality. Having a chronic disease was associated with increased likelihood of distancing in public, but not of staying home or masking, despite the increased risk of COVID-19 morbidity and mortality resulting among this group [38]. This demonstrates a need for interventional targeting of women living with chronic diseases. Fear of COVID-19 only influenced physical distancing in public as a prevention behavior, and only minimally (8% increased odds); our findings suggest that fear is not an effective tactic for uptake of COVID-19 prevention behaviors, and should not be employed in interventions, though other literature suggests fear can be an effective tactic [13,39]. Given the mixed findings, as well as the potential for adverse mental health associated with fear of COVID-19 [40], we would not recommend the use of fear as a public health messaging tactic relating to COVID-19. Knowing someone who had been diagnosed with COVID-19 was significant at the bivariate level for all behaviors; however, it was only retained in Model 2 for staying home; a three-fold increase in odds was observed. This may be reflective of minimal perceived susceptibility, including the perception of oneself as behaving in a more “safe” manner than others, or minimal perceived severity based on disease course of those known to have had COVID-19 (i.e., mild symptoms and morbidity). Further, this could be influenced by who the known individual diagnosed with COVID-19 is; diagnosis of a family member may increase perceived susceptibility and practice of prevention behaviors more than diagnosis of a coworker. Further insight is needed on this, particularly for use of increased awareness of COVID-19 in one’s social network.

### 4.4. Organizational Context

Relationship status was impactful for staying home and physical distancing in public. Being in a nonmarriage committed relationship increased the odds of staying home by over seven-fold, though being married did not impact this behavior. Understanding the differing demands on a marriage than a nonmarriage committed relationship, including children, higher household occupancy, greater need for leaving the home for household needs, or crowding, may offer interventional insights. For physical distancing, any committed relationship led to 2–2.5 times increased odds, though only significantly among married participants. Potential alterable mechanisms for this include altruism towards a partner or social pressure and/or validation to practice prevention behaviors if a partner is practicing them.

Annual household income greatly impacted the likelihood of staying home, with multiple-fold increases in the odds of practicing of each behavior in households making USD <30,000 a year, and households making USD >50,000 a year, compared to households making USD 30,000–50,000. It is possible that these represent two distinct groups, practicing behaviors for divergent reasons. Those within the lowest income group are more likely to be essential workers [41], who may acknowledge an already increased work-related risk. This would indicate that risk/exposure education is a potential effective intervention. Those in households making greater than USD 50,000 a year are likely to have greater health literacy [36], increasing the likelihood of uptake of prevention activities. Those within the middle-income group may have less risk at work, but less health literacy or access to health education to promote precautionary behaviors.

### 4.5. Environmental Context

Those living in urban environments were 58% less likely to stay home, 45% less likely to physically distance in public, and approximately 60% less likely to wear a face mask than women living in nonurban environments. Urban residents face additional challenges to practicing some prevention behaviors: overcrowding [42], making staying at home more difficult, and higher population density in urbans areas, leading to challenges in maintaining physical distancing in public [43]. Urban residents may be more likely to work in service industry jobs or face financial insecurity [44], necessitating leaving the home for essential or nonessential work. Further, lower income may lead to financial inability to purchase masks, and use of less effective or no face coverings. Urban environments are most susceptible to outbreaks of COVID-19, due to movement in and out of cities for work and/or leisure [45]. Given these findings and possible barriers to preventative behaviors, elucidation of mechanisms of COVID-19 prevention behaviors is vital, as is interventional focus on urban environments.

## 5. Conclusions

The primary limitations of our research are the use of nonprobability sampling methods and self-reported measures. Only women with access to the internet were able to access the survey, and most advertisements ran on Facebook; however, 75% of U.S. adult women use Facebook, of whom three-quarters accessing the site daily [46], indicating that the sampling frame encompassed most U.S. women. Compared to the U.S. population as of 2019, our sample approximates the population non-Hispanic Black women (12.2% vs. 12.8%), underrepresents Hispanic/Latinx women (9.8% vs. 18.0%), and overrepresents API (13.0% vs. 6.1%) and AI/AN (5.5% vs. 0.7%) women in the U.S. as of 2019 [47]; given the high burden of COVID among API and AI/AN populations, such overrepresentation may add to the utility of the current findings; however, the underrepresentation of Hispanic/Latinx women is notable and of concern given the high burden of COVID and representation in the essential workforce. These findings may not be generalizable to women in rural areas of the U.S., as the current sample slightly underrepresents the 19.3% of the U.S. population in rural areas [48]. Women in The COPE Study were on average 35 years old, and therefore may not represent women aged 50 and older. Dichotomous response options for practice of prevention behaviors limit the ability to understand frequency of the behavior, and may result in an overestimate of “compliance” with the prevention behavior. However, pragmatically, any practice of prevention behavior is a more positive outcome than no practice, supporting the potential significance of this analysis for informing interventions and health education. As previously acknowledged, race, and depending on context, educational attainment, do not dictate prevention behavior, but rather may be associated with prevention behavior due to social conditions of discrimination and classism. Analysis and conclusions should be interpreted with this in mind. Finally, this paper presents results from only one month of an ongoing pandemic, which has seen multiple resurgences and changes to policy surrounding prevention activities, though notably the prevention activities detailed here have remained constant. Follow-up data collection is currently ongoing, with the intention to disseminate comparative finding over the course of the COVID-19 pandemic.

Despite the limitations of the current data, we believe that the findings include valuable information for promotion of prevention behaviors in the COVID-19 pandemic and in future epidemics. Practice of prevention behaviors is complex and multifaceted, with no single “silver bullet” interventional target. Each prevention behavior requires unique considerations for tailoring to different populations. Staying home may not be feasible for all women but provides the most protection. Physical distancing can be promoted by visual cues and limits on space density [3], but is limited by the availability of space and compliance of other individuals in ones’ surroundings. Both distancing and masking can be promoted through social cues but can likewise be discouraged. Given the placement of women as modelers of behavior in familial and community contexts, it may be highly impactful to engage them in interventions aiming to set social standards for prevention behaviors. Race/ethnicity, education level, annual household income, and urban residence were the strongest factors identified for interventional targeting of U.S. women. Several potential interventional “levers” are suggested to facilitate this. Efforts to increase knowledge around testing availability and accessibility, as well as campaigns emphasizing interpersonal relationships with those known to have had COVID-19 and those at-risk for COVID-19 may prove impactful for increasing preventative behavior. Further, interventions to increase ease of staying home, such as food delivery support and remote working should be promoted, while adaptation of public space to facilitate and promote physical distance should be continued and enhanced. Governmental engagement in these processes is crucial. Public health prevention efforts and funding should appropriately consider the needs of U.S. women with attention to these factors.

## Figures and Tables

**Table 1:** Characteristics of women enrolled in The COPE Study.

			*n* (%) or Median (IQR)
Personal	Race/Ethnicity	American Indian or Alaskan Native (AI/AN)	27 (5.5)
		Asian or Pacific Islander (API)	64 (13.0)
		Black	60 (12.2)
		Latinx	48 (9.8)
		White	241 (49.1)
		Other or Multiracial	33 (6.7)
	Education	Completed High School (HS) or equivalent (GED), or less	91 (18.5)
		Some trade or vocational school, or some college	78 (15.9)
		Completed trade or vocational school or college	181 (36.9)
		Some or completed graduate school	122 (24.8)
	Employment	Employed	304 (61.9)
		Unemployed Prior to 1 March	119 (24.2)
		Become Unemployed Since 1 March 2020	68 (13.8)
	Age (Years)		33 (28, 40)
	No. Children Under 18		0 (0, 1) 0.59 (1.1) *
	No. Children 18 or Older		0 (0, 0) 0.42 (1.2) *
	Have a Chronic Disease		117 (23.8)
	Had COVID-19 Symptoms		77 (15.7)
	Been Tested for COVID-19		28 (5.7)
	Know where to get tested for COVID-19		23 (46.8)
	Diagnosed with COVID-19		8 (1.6)
	Fear of COVID Scale		22 (16, 26)
Interpersonal	Relationship Type	Not Partnered	221 (45.0)
		Committed, not Married	100 (20.4)
		Married	170 (34.6)
	Know someone who had COVID-19		83 (16.9)
	Know someone hospitalized for COVID-10		35 (7.1)
	Know someone who has died from COVID-19		18 (3.7)
Organizational	Living Alone		213 (43.4)
	Household size		2 (1, 3)
	No. Children under 18 staying in household		2 (0, 3)
	No. Children 18 or older staying in household		3 (2, 4)
	Annual Household Income	USD < 30,000	141 (28.7)
		USD 30,000–50,000	118 (24.0)
		USD > 50,000	196 (39.9)
Environmental	Region	West	142 (28.9)
		Midwest	100 (20.4)
		South	155 (31.6)
		Northeast	80 (16.3)
	Community Environment	Urban	216 (44.0)
		Suburban	176 (35.8)
		Rural	83 (16.9)
Outcome Variables	Staying home except for essential activities		428 (87.2)
	Physical distancing in public		418 (85.1)
	Wearing a face mask in public		386 (78.6)

Totals may not add up to 100% due to missing data. * Mean (SD) is presented for meaningfulness.

**Table 2:** Bivariate relationships between independent and outcome variables.

	COVID-19 Prevention Behaviors						
		Staying Home		Physical Distancing		Wearing a Face Mask	
		*n* (%)	*p*	*n* (%)	*p*	*n* (%)	*p*
Personal	Race/Ethnicity						
	AI/AN	24 (88.9)	**0.051**	21 (77.8)	**0.092**	20 (74.1)	0.579
	API	58 (90.6)		59 (92.2)		51 (79.7)	
	Black	52 (86.7)		52 (86.7)		48 (80.0)	
	Latinx	36 (75.0)		36 (75.0)		34 (70.8)	
	White	220 (91.3)		212 (88.0)		196 (81.3)	
	Other or Multiracial	29 (87.9)		29 (87.9)		28 (84.8)	
	Education						
	Completed HS or GED, or less	76 (83.5)	**0.200**	77 (84.6)	**0.004**	61 (67.0)	**<0.001**
	Some trade/vocational school or college	73 (93.6)		74 (94.9)		68 (87.2)	
	Completed trade/vocational school or college	161 (89.0)		147 (81.2)		140 (77.3)	
	Some/completed graduate school	110 (90.2)		113 (92.6)		110 (90.2)	
	Employment Status						
	Employed	263 (86.5)	**0.164**	259 (85.2)	0.879	246 (80.9)	**0.022**
	Unemployed Prior to 1 March	101 (84.9)		100 (84.0)		83 (69.7)	
	Unemployed since 1 March 2020	64 (94.1)		59 (86.8)		57 (83.8)	
	Age (Years)						
	Practicing Behavior	35.3 (11.2)	**0.136**	35.4 (11.0)	0.919	35.7 (11.3)	0.943
	Not Practicing Behavior	36.2 (9.0)		35.0 (10.6)		34.3 (9.3)	
	No. Children Under 18						
	Practicing Behavior	0.61 (1.1)	0.726	0.63 (1.1)	0.285	0.62 (1.1)	**0.220**
	Not Practicing Behavior	0.61 (1.0)		0.5 (1.0)		0.55 (1.1)	
	No. Children 18 or Older						
	Practicing Behavior	0.45 (1.2)	0.608	0.39 (1.0)	0.305	0.48 (1.3)	**0.052**
	Not Practicing Behavior	0.31 (1.0)		0.69 (2.1)		0.26 (0.9)	
	Have a Chronic Disease						
	Yes	107 (91.5)	0.417	108 (92.3)	**0.054**	100 (85.5)	**0.139**
	No	317 (88.8)		305 (85.4)		282 (79.0)	
	Had COVID-19 Symptoms						
	Yes	71 (92.2)	**0.15**	67 (87.0)	0.613	59 (76.6)	0.643
	No	357 (86.2)		351 (84.8)		327 (79.0)	
	Been Tested for COVID-19						
	Yes	27 (96.4)	**0.237**	24 (85.7)	1	22 (78.6)	1
	No	402 (86.6)		394 (85.1)		364 (78.6)	
	Know where to get tested for COVID-19						
	Yes	208 (90.4)	**0.042**	208 (90.4)	**0.002**	200 (87.0)	**<0.001**
	No or Unsure	220 (84.3)		210 (80.5)		186 (71.3)	
	Diagnosed with COVID-19						
	Yes	6 (75.0)	0.274	5 (62.5)	**0.101**	5 (62.5)	0.377
	No	422 (87.4)		413 (85.5)		381 (78.9)	
	Fear of COVID Scale						
	Practicing Behavior	21.4 (6.7)	**0.005**	21.3 (6.5)	**0.072**	21.2 (6.5)	0.451
	Not Practicing Behavior	18.5 (7.2)		19.6 (8.9)		20.7 (8.3)	
Interpersonal	Relationship Type						
	Not Partnered	183 (82.8)	**0.005**	175 (79.2)	**0.004**	257 (71.0)	**0.001**
	Committed, not Married	96 (96.0)		90 (90.0)		85 (85.0)	
	Married	149 (87.6)		153 (90.0)		144 (84.7)	
	Know someone who had COVID-19						
	Yes	78 (94.0)	**0.042**	77 (92.8)	**0.032**	70 (84.3)	**0.163**
	No	350 (85.8)		341 (83.6)		316 (7.5)	
	Know someone hospitalized for COVID-19						
	Yes	33 (94.3)	**0.292**	32 (91.4)	0.457	29 (82.9)	0.525
	No	395 (86.6)		386 (84.6)		357 (78.3)	
	Know someone who has died from COVID-19						
	Yes	18 (100.0)	**0.148**	17 (94.4)	0.495	16 (88.9)	0.387
	No	410 (86.7)		401 (84.8)		370 (78.2)	
Organizational	Living with Others						
	Yes	238 (92.6)	**0.005**	221 (86.0)	0.566	210 (81.7)	0.370
	No	180 (84.5)		187 (87.8)		167 (78.4)	
	No. Children Under 18 staying in household						
	Practicing Behavior	0.54 (1.2)	**0.227**	0.54 (1.2)	**0.074**	0.52 (1.2)	0.264
	Not Practicing Behavior	0.32 (0.9)		0.38 (1.2)		0.5 (1.3)	
	No. Children 18 or older staying in household						
	Practicing Behavior	0.11 (0.5)		0.09 (0.5)		0.1 (0.5)	
	Not Practicing Behavior	0.14 (0.8)	0.625	0.23 (1.0)	0.6	0.1 (0.7)	**0.163**
	Annual Household Income						
	USD <30,000	130 (92.2)		126 (89.4)		114 (80.9)	
	USD 30,000–50,000	93 (78.8)		92 (78.0)		82 (69.5)	
	USD >50,000	179 (91.3)	**0.001**	176 (89.8)	**0.006**	171 (87.2)	**0.001**
Environmental	Region						
	West	126 (88.7)		124 (87.30		107 (75.4)	
	Midwest	86 (86.0)		86 (86.0)		74 (74.0)	
	South	138 (88.5)		135 (86.5)		131 (84.0)	
	Northeast	74 (92.5)	0.596	70 (87.5)	0.988	71 (88.8)	**0.023**
	Community Environment						
	Urban	181 (83.8)		176 (81.5)		159 (73.6)	
	Suburban	164 (93.2)		161 (91.5)		151 (85.8)	
	Rural	77 (92.8)	**0.006**	74 (89.2)	**0.012**	71 (85.5)	**0.004**

Bold font variables are significant at the level of *p* < 0.250.

**Table 3:** Binary logistic regressions for outcome 1, staying home.

			Model 2 *n* = 413; *p* < 0.10		Model 3 *n* = 421; *p* < 0.05	
			Exp (B)	*p*	Exp (B)	*p*
Personal	Race/Ethnicity (Reference: White)	AI/AN	0.409	0.239	0.553	0.414
		API	0.588	0.365	0.753	0.606
		Black	0.664	0.467	0.872	0.804
		Latinx	0.220	0.003	0.287	0.011
		Other/Multiple	0.323	0.112	0.424	0.194
	Age (Years)		0.971	0.073		
	Fear of COVID Scale		1.079	0.004	1.075	0.005
Interpersonal	Relationship status (Reference: not partnered)	Committed, not married	6.819	0.015	7.095	0.012
		Married	0.768	0.514	0.771	0.509
	Know someone who had COVID-19		2.986	0.071		
	Annual household income (Reference: USD 30,000–50,000)	<30,000	4.725	0.001	4.317	0.001
		>50,000	3.473	0.004	3.495	0.003
Environmental	Community environment (Reference: nonurban)	Urban	0.400	0.017	0.421	0.021
	Cox and Snell Pseudo-R^2^		0.124		0.109	
	Nagelkerke Pseudo-R^2^		0.252		0.221	
	Model *p*-value		<0.001		<0.001	

**Table 4:** Binary logistic regressions for outcome 2, physically distancing six feet.

			Model 2 *p* < 0.10; *n* = 413		Model 3 *p* < 0.05; *n* = 450	
			Exp (B)	*p*	Exp (B)	*p*
Personal	Race/Ethnicity (Reference: White)	AI/AN	0.655	0.470	0.585	0.339
		API	4.323	0.027	3.632	0.047
		Black	1.762	0.262	1.546	0.378
		Latinx	0.556	0.201	0.470	0.092
		Other	1.265	0.703	1.152	0.815
	Education (Reference: High School Diploma, GED, or less)	Some trade or vocational school, or some college	3.983	0.028	4.044	0.025
		Completed trade or vocational school or college	0.753	0.474	0.767	0.497
		Some or completed graduate school	2.731	0.051	2.626	0.058
	Have a chronic disease		3.103	0.012	3.334	0.008
	Diagnosed with COVID-19		0.068	0.004	0.052	0.002
Interpersonal	Relationship status (Reference: not partnered)	Committed, not married	2.001	0.096	1.924	0.114
		Married	2.285	0.036	2.662	0.011
Community	Community environment (Reference: nonurban)	Urban	0.522	0.054		
	Cox and Snell Pseudo-R^2^		0.104		0.093	
	Nagelkerke Pseudo-R^2^		0.196		0.177	
	Model *p*-value		<0.001		<0.001	

**Table 5:** Binary logistic regressions for outcome 3, wearing a face mask in public.

			Model 2 *n* = 439; *p* < 0.10		Model 3 *n* = 439; *p* < 0.05	
			Exp (B)	*p*	Exp (B)	*p*
Personal	Education (Reference: High School Diploma, GED, or less)	Some trade or vocational school, or some college	3.455	0.007	3.562	0.005
		Completed trade or vocational school or college	1.598	0.157	1.573	0.161
		Some or completed graduate school	4.435	0.001	4.454	0.001
	Know where to get tested for COVID-19		1.967	0.014	2.00	0.010
Organizational	Annual household income (Reference: USD 30,000–50,000)	<30,000	2.284	0.016	2.156	0.022
		>50,000	2.25	0.013	2.184	0.013
Environmental	Region (Reference: Northeast)	Midwest	0.442	0.007		
		South	1.019	0.968		
		West	0.646	0.321		
	Community environment (Reference: nonurban)	Urban	0.433	0.003	0.41	0.002
	Cox and Snell Pseudo-R^2^		0.114		0.102	
	Nagelkerke Pseudo-R^2^		0.184		0.165	
	Model *p*-value		<0.001		<0.001	

## Data Availability

The data presented in this study are available on request from the corresponding author. The data are not publicly available due to the sensitive nature of data collection and confidentiality concerns.

## Supplementary Materials

The following are available online at https://www.mdpi.com/1660-4601/18/1/180/s1, Table S1: Binary Logistic Regressions for Outcome 1, Staying Home, Table S2: Binary Logistic Regressions for Outcome 2, Physical Distancing 6 Feet While in Public, Table S3: Binary Logistic Regressions for Outcome 3, Wearing a Face Mask in Public.
