# Supplementary material for: Staying Home, Distancing, and Face Masks: COVID-19 Prevention among U.S. Women in The COPE Study
Source: Int J Environ Res Public Health. 2020 Dec 29;18(1):180. doi: 10.3390/ijerph18010180 (PMC7795406; doi:10.3390/ijerph18010180)
Supplement: Supplementary file 1 [file ijerph-18-00180-s001.pdf]

Supplementary Table 1. Binary Logistic Regressions for Outcome 1, Staying Home

			**Model 1**		**Model 2 ***		**Model 3 ****	
			***N* = 407**		***N* = 413**		***N* = 421**	
			**Exp (B)**	***p***	**Exp (B)**	***p***	**Exp (B)**	***p***
Personal	Race/Ethnicity (Reference: White)	AI/AN	0.455	0.316	0.409	0.239	0.553	0.414
		API	0.636	0.474	0.588	0.365	0.753	0.606
		Black	0.622	0.427	0.664	0.467	0.872	0.804
		Latinx	0.356	0.068	0.220	0.003	0.287	0.011
		Other/Multiple	0.318	0.118	0.323	0.112	0.424	0.194
	Education (Reference: High School Diploma, GED, or less)	Some trade or vocational school, or some college	1.684	0.421				
		Completed trade or vocational school or college	1.796	0.238				
		Some or completed graduate school	2.061	0.195				
	Unemployment (Reference: Employed)	Since March 1, 2020	1.817	0.410				
		Unemployed Prior to March 1, 2020	1.369	0.539				
	Age (Years)		0.980	0.238	0.971	0.073		
	Has had COVID symptoms		0.703	0.518				
	Know where to get tested for COVID-19		1.071	0.858				
	Has been tested for COVID-19		2.866	0.364				
	Fear of COVID Scale		1.075	0.010	1.079	0.004	1.075	0.005
Interpersonal	Relationship status (Reference: not partnered)	Committed, not married	5.498	0.034	6.819	0.015	7.095	0.012
		Married	0.564	0.214	0.768	0.514	0.771	0.509
	Know someone who had COVID-19		2.306	0.184	2.986	0.071		
Organizational	Living with others		1.700	0.163				
	Annual household income (Reference: $30,000-$50,000)	<$30,000	3.959	0.009	4.725	0.001	4.317	0.001
		>$50,000	3.117	0.014	3.473	0.004	3.495	0.003
	No. Children under 18 staying in household		1.482	0.113				

Community	Community environment (Reference: non-urban)	Urban	0.409	0.027	0.400	0.017	0.421	0.021
	Cox & Snell Pseudo-R^2		0.124		0.124		0.109	
	Nagelkerke Pseudo-R^2		0.256		0.252		0.221	
	Model p-value		<0.001		<0.001		<0.001	

* Variables in model are significant at $p < 0.10$; ** Variables in model are significant at $p < 0.05$

Supplementary Table 2. Binary Logistic Regressions for Outcome 2, Physical Distancing 6 Feet While in Public

			**Model 1**		**Model 2 ***		**Model 3 ****	
			***N* = 407**		***N* = 413**		***N* = 450**	
			**Exp (B)**	***p***	**Exp (B)**	***p***	**Exp (B)**	***p***
Personal	Race/Ethnicity (Reference: White)	AI/AN	0.735	0.625	0.655	0.470	0.585	0.339
		API	4.487	0.029	4.323	0.027	3.632	0.047
		Black	1.431	0.505	1.762	0.262	1.546	0.378
		Latinx	0.633	0.379	0.556	0.201	0.470	0.092
		Other	1.450	0.577	1.265	0.703	1.152	0.815
	Education (Reference: High School Diploma, GED, or less)	Some trade or vocational school, or some college	4.109	0.053	3.983	0.028	4.044	0.025
		Completed trade or vocational school or college	0.655	0.340	0.753	0.474	0.767	0.497
		Some or completed graduate school	3.002	0.059	2.731	0.051	2.626	0.058
	Have a chronic disease		3.265	0.020	3.103	0.012	3.334	0.008
	Know where to get tested for COVID-19		1.308	0.446				
	Diagnosed with COVID-19		0.050	0.002	00.068	0.004	0.052	0.002
Interpersonal	Fear of COVID Scale		1.034	0.171				
	Relationship status (Reference: not partnered)	Committed, not married	2.082	0.126	2.001	0.096	1.924	0.114
		Married	1.952	0.127	2.285	0.036	2.662	0.011
	Know someone who has had COVID-19		2.013	0.202				
Organ	No. children under 18 staying in household		1.273	0.201				
		<$30,000	1.943	0.141				

			Exp (B)	p	Exp (B)	p	Exp (B)	p
	Annual household income (Reference: $30,000-$50,000)	>$50,000	1.723	0.190				
Community	Community environment (Reference: non-urban)	Urban	0.512	0.065	0.522	0.054		
	Cox & Snell Pseudo-R^2		0.136		0.104		0.093	
	Nagelkerke Pseudo-R^2		0.254		0.196		0.177	
	Model p-value		<0.001		<0.001		<0.001	

* Variables in model are significant at $p < 0.10$; ** Variables in model are significant at $p < 0.05$

Supplementary Table 3: Binary Logistic Regressions for Outcome 3, Wearing a Face Mask in Public

			**Model 1** $N = 421$		**Model 2 *** $N = 439$		**Model 3 **** $N = 439$	
			Exp (B)	*p*	**Exp (B)**	*p*	**Exp (B)**	*p*
Personal	Education (Reference: High School Diploma, GED, or less)	Some trade or vocational school, or some college	3.833	0.005	3.455	0.007	3.562	0.005
		Completed trade or vocational school or college	1.766	0.115	1.598	0.157	1.573	0.161
		Some or completed graduate school	5.038	0.001	4.435	0.001	4.454	0.001
	Unemployment (Reference: Employed)	Since March 1, 2020	1.106	0.819				
		Unemployed Prior to March 1, 2020	0.657	0.232				
	No. children under 18		0.936	0.636				
	No. children 18 and Older		1.451	0.088				
	Have a chronic disease		1.448	0.295				
	Know where to get tested for COVID-19		1.705	0.072	1.967	0.014	2.00	0.010
Interpersonal	Relationship status (Reference: Not Partnered)	Committed, not married	1.750	0.139				
		Married	1.339	0.405				
	Know someone who had COVID-19		0.996	0.993				
Organiz	No. children 18 and older staying in household		0.877	0.693				

	Annual household income Reference: $30,000-$50,000)	<$30,000	2.745	0.009	2.284	0.016	2.156	0.022
		>$50,000	2.479	0.010	2.25	0.013	2.184	0.013
Community	Region (Reference: Northeast)	Midwest	0.390	0.055	0.442	0.007		
		South	1.026	0.958	1.019	0.968		
		West	0.710	0.466	0.646	0.321		
	Community environment (Reference: Non-urban)	Urban	0.408	0.003	0.433	0.003	0.41	0.002
	Cox & Snell Pseudo-R^2		0.137		0.114		0.102	
	Nagelkerke Pseudo-R^2		0.223		0.184		0.165	
	Model *p*-value		<0.001		<0.001		<0.001	

* Variables in model are significant at $p < 0.10$; ** Variables in model are significant at $p < 0.05$
